# Genotype-Specific Postural Control Deficits in Hemophilia A: Insights from Center of Pressure Analysis Beyond Radiographic Arthropathy

**DOI:** 10.3390/ijms27052323

**Published:** 2026-03-01

**Authors:** Ya-Chi Huang, Wei-Long Wang, Hsuan-Yu Lin, Peng-Ta Liu, Cheng-Wei Huang, Ming-Ching Shen, Ming Chen, Shun-Ping Chang, Adeline Yan, Shao-Li Han

**Affiliations:** 1 Department of Physical Medicine and Rehabilitation, Changhua Christian Hospital, Changhua 500, Taiwan; 184970@cch.org.tw; 2 Division of General Practice, Department of Medical Education, Changhua Christian Hospital, Changhua 500, Taiwan; tmuleo880819@gmail.com; 3 Division of Hematology/Oncology, Department of Internal Medicine, Changhua Christian Hospital, Changhua 500, Taiwan; 93687@cch.org.tw (H.-Y.L.); 181014@cch.org.tw (C.-W.H.); 4 Department of Senior Wellness and Sport Science, Tunghai University, Taichung 407, Taiwan; ethan_liupt@thu.edu.tw; 5 Department of Senior Health and Exercise Science, Tunghai University, Taichung 407, Taiwan; 6 Hemophilia Treatment and Thrombosis Center, Department of Internal Medicine, Changhua Christian Hospital, Changhua 500, Taiwan; 7 Department of Internal Medicine, National Taiwan University Hospital, Taipei 100, Taiwan; 8 Department of Laboratory Medicine, National Taiwan University Hospital, Taipei 100, Taiwan; 9 Department of Genomic Medicine, Changhua Christian Hospital, Changhua 500, Taiwan; 104060@cch.org.tw (M.C.); 70914@cch.org.tw (S.-P.C.); 181435@cch.org.tw (A.Y.); 10 Department of Obstetrics and Gynecology, Changhua Christian Hospital, Changhua 500, Taiwan; 11 Department of Medical Genetics, National Taiwan University Hospital, Taipei 100, Taiwan; 12 Department of Obstetrics and Gynecology, National Taiwan University Hospital, Taipei 100, Taiwan; 13 Department of Physical Medicine and Rehabilitation, Kaohsiung Show Chwan Memorial Hospital, Kaohsiung 821, Taiwan

**Keywords:** hemophilia, genetic mutations, center of pressure, postural control

## Abstract

Hemophilia is an X-linked inherited bleeding disorder characterized by joint hemorrhages and progressive arthropathy. While mutation type is known to influence disease severity, its impact on postural balance strategies has remained unclear. This cross-sectional study investigated the relationship between gene mutation type and postural control in hemophilia A patient using center of pressure (CoP) analysis and radiographic joint assessment with the Pettersson score. Thirty-five participants were divided into an INV group (intron 22 or intron 1 inversion of the *F8* gene) and a NonINV group (other mutations). While the Pettersson scores and traditional time-domain CoP parameters (sway area, velocity) were comparable between groups, frequency domain analysis revealed a significant difference. INV group exhibited significantly higher energy content above 2 Hz in the anteroposterior direction compared to NonINV group. This genotype-specific spectral signature emerged despite comparable radiographic arthropathy and conventional CoP metrics, suggesting that frequency-domain CoP analysis can uncover subclinical postural adaptations in hemophilia A. These findings highlight the need for targeted proprioceptive training in this specific subpopulation to prevent subclinical instability and potential falls.

## 1. Introduction

Hemophilia is an X-linked recessive bleeding disorder caused by deficiencies in clotting factors VIII or IX. This condition results in recurrent bleeding, even after minor trauma, and it mostly affects synovial joints due to their nature of motion, which makes them prone to injury [1,2,3]. Over 80% of bleeding episodes occur in weight-bearing joints such as knees and ankles, leading to chronic synovitis, cartilage degeneration, and debilitating hemophilic arthropathy [1]. Despite advances in prophylactic therapies, subclinical bleeding and progressive joint damage persist in many patients, resulting in lifelong mobility impairments and reduced quality of life [4,5].

The clinical heterogeneity of hemophilia is closely linked to genetic mutation types [6,7,8]. Chen et al. have documented that intron 22 inversions in *F8* are associated with an increased risk of severe arthropathy [9]. Furthermore, large deletions and nonsense variants contribute to the complete loss of coagulation factor VIII [10]. Major deficit in coagulation function predisposes subjects with hemophilia to recurrent joint bleeding, resulting in chronic synovitis and more severe functional impairments [11]. These findings suggest that genetic mutation types may influence the risk of developing arthropathy and the progress of arthropathy due to various degrees of coagulation deficiency.

The way to evaluate joint arthropathy nowadays relies mainly on anatomic imaging, including, traditionally, X-ray films, ultrasonography and Magnetic resonance imaging (MRI). The radiographic X-rays can detect anatomic changes, though always in the later stage of osteochondral damage, with the advantage of availability, affordability, and time efficiency. MRI can identify intra-articular bleeding and early stages of synovial hypertrophy [12]. However, MRI is more expensive and has less availability compared with other image tools. In contrast, ultrasound has been proven efficacious as a useful monitor tool for joint bleeding and progress of arthropathy [13]. These image tools are valuable for structural changes. However, these anatomical structures do not always correlate with limitations in daily activities. The Pettersson score is used for long-term follow-up of joint damage progression in patients. Increasing scores suggest worsening of joint pathology, which is crucial for guiding treatment strategies. Higher Pettersson scores are typically associated with poorer functional outcomes, potentially requiring more aggressive physical therapy or consideration of surgical interventions. Since the Pettersson score is based on X-ray evaluations, it may not adequately detect early soft tissue changes such as synovitis, which limits its sensitivity to early hemophilic arthropathy. Additionally, the subjective nature of scoring can lead to variability between different clinicians [12,14]. To address this limitation and achieve a comprehensive evaluation of joint health, gait analysis provides an alternative way to address these challenges [2,15,16].

Gait analysis is adopted by using optical markers and weight sensors to evaluate joint motion while standing or walking [17]. It provides subjective and numerical data, which is good for following specific disease conditions. Three-dimensional gait analysis and postural assessment, especially the center of pressure (CoP) measurements, have been proven as an efficacious approach for early balance deficits and altered weight distribution strategies in hemophilia patients [18,19,20,21,22]. CoP parameters also reveal reduced mediolateral stability and delayed proprioceptive responses even in radiographically normal joints. These approaches provide additional insights into joint functionality, which are complementary for structural imaging and standard clinical functional scores. However, there has been a study gap between gene mutations and clinical postural assessment.

This study aims to explore the relationship between specific genetic variants and hemophilic arthropathy, with a focus on their impact on postural control. We utilize the CoP analysis in both the time and frequency domains to investigate the association between genetic types and postural control [20]. Furthermore, we hypothesize that individuals with hemophilia who carry *INV22* gene mutation may demonstrate more pronounced impairments in balance control, potentially due to disruption of the *F8* gene, resulting in absent or nonfunctional factor VIII and causing a severe bleeding phenotype, characterized by frequent spontaneous joint and muscle bleeds and very low (<1%) factor VIII activity [8,23,24]. Conversely, the null hypothesis posits that there are no significant differences in postural control strategies, as measured by CoP parameters, between hemophilia A patients with the intron 22/1 inversion genotype (INV) and those with non-inversion mutations (NonINV).

## 2. Results

### 2.1. Study Population

A total of 83 individuals were screened, with 35 participants enrolled after applying exclusion criteria. To ensure a homogenous analysis, participants diagnosed with hemophilia B (*n* = 12) were excluded from this study because of their distinct genetic etiologies, and the mutation spectra differ in frequency and type [25,26]. The other 36 non-enrolled participants were primarily excluded due to missed follow-up visits, transportation issues, or other personal factors, which hindered continued participation and data collection. Participants with hemophilia A were further categorized into two groups based on the gene variants: Group A with inversion 22 and inversion 1 and Group B with variant types other than inversion type. The demographic characteristics of the study population are presented in Table 1. (Detailed personal information is presented in Supplementary Table S1).

The descriptive statistics revealed that Group A (*n* = 16) had a mean Pettersson lower limbs score of 9.44 with a standard deviation of 12.34, while Group B (*n* = 19) had a slightly lower mean score of 8.11 and a standard deviation of 8.74. To examine whether this difference reaches a statistical significance, we adopt a Mann–Whitney *U* test. The results revealed that a *U* statistic of 150.0 and a *p*-value of 0.96 (*p* > 0.05). The difference in Pettersson lower limbs score between the two groups is not considered statistically significant. Furthermore, the effect size, as measured by Cohen’s *d* (0.13), indicates a trivial difference in the magnitude of scores between Group A and Group B. Additional details of Pettersson score of lower limbs in individual participants are presented in Supplementary Table S2.

### 2.2. Mutation Type in Hemophilia

The gene variants of the two groups are detailed in Table 2.

### 2.3. Postural Analysis

Among the 72 commonly analyzed parameters derived from CoP data, representative variables were selected and categorized into four functional domains—positional, dynamic, frequency, and stochastic—based on their clinical relevance and statistical significance [26]. The selection strategy prioritized parameters with statistically significant between-group differences or those commonly cited in the literature as key indicators of postural stability.

**Figure 1:**
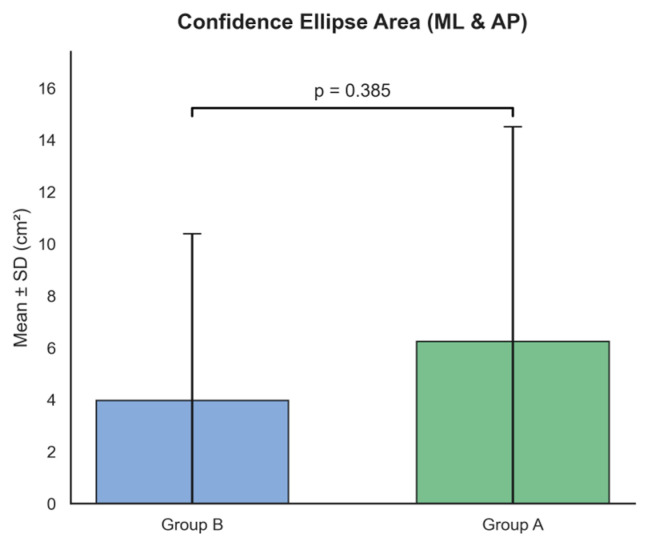
Comparison of positional features between Group A and Group B. The bar plot illustrates the 95% Confidence Ellipse Area (ML & AP) in cm^2^. No significant inter-group difference was observed (*p* = 0.385), suggesting that the spatial extent of static postural sway is preserved across cohorts. Data are expressed as Mean ± SD.

In the positional domain (Figure 1), the confidence ellipse area (confidence_ellipse_area_ML_AND_AP) did not significantly differ between groups after adjusting for age (Group A: 6.24 ± 8.26 cm^2^ vs. Group B: 3.96 ± 6.42 cm^2^, *p* = 0.385), suggesting comparable static stability between the two cohorts. This parameter was included due to its frequent use in prior studies as a general measure of postural stability.

**Figure 2:**
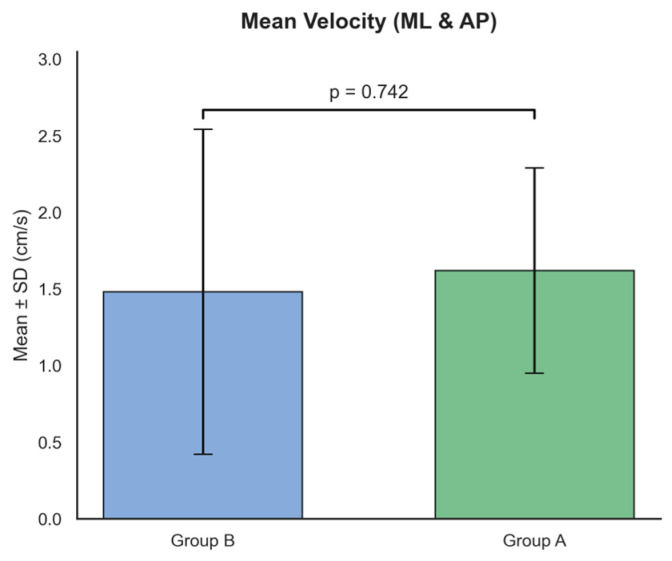
Comparison of dynamic features between Group A and Group B. The bar plot represents the Mean Velocity (ML & AP) measured in cm/s. No statistical significance was reached (*p* = 0.742), indicating that the velocity-based characteristics of postural sway are similar between Group A and Group B. Data are expressed as Mean ± SD.

In the dynamic domain (Figure 2), mean velocity (mean_velocity_ML_AND_AP), recognized as a reliable indicator of fall risk, was examined. The results showed no statistically significant difference between the groups (Group A: 1.62 ± 0.67 cm/s vs. Group B: 1.48 ± 1.06 cm/s, *p* = 0.742). Similarly, other dynamic measures were comparable between groups, indicating that the overall speed of postural adjustments remains preserved across different mutation types [27].

**Figure 3:**
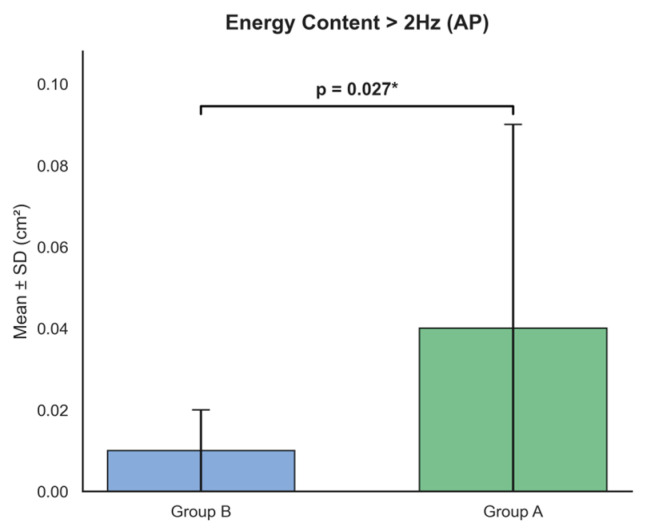
Comparison of frequency features between Group A and Group B. A statistically significant increase in Energy Content > 2 Hz (AP) was observed in Group A relative to Group B (*p* = 0.027), highlighting a distinct frequency-domain signature in the anteroposterior axis. Data are expressed as Mean ± SD in cm^2^. The asterisk (*) indicates a statistically significant difference between groups (*p* < 0.05).

In the frequency domain (Figure 3), energy content above 2 Hz in the anteroposterior direction (energy_content_above_2_Power_Spectrum_Density_AP) showed a significant difference, with significantly higher values in Group A (Group A: 0.04 ± 0.05 cm^2^ vs. Group B: 0.01 ± 0.01 cm^2^, *p* = 0.027). This corresponds to a positive beta coefficient (*β* = 0.029), indicating that Group A participants exhibited increased high-frequency sway components in the anteroposterior direction after controlling for age. In contrast, the frequency mode in the mediolateral direction (frequency_mode_Power_Spectrum_Density_ML), which was noted in previous studies, did not reach statistical significance in this cohort (Group A: 0.22 ± 0.10 Hz vs. Group B: 0.25 ± 0.09 Hz, *p* = 0.325).

**Figure 4:**
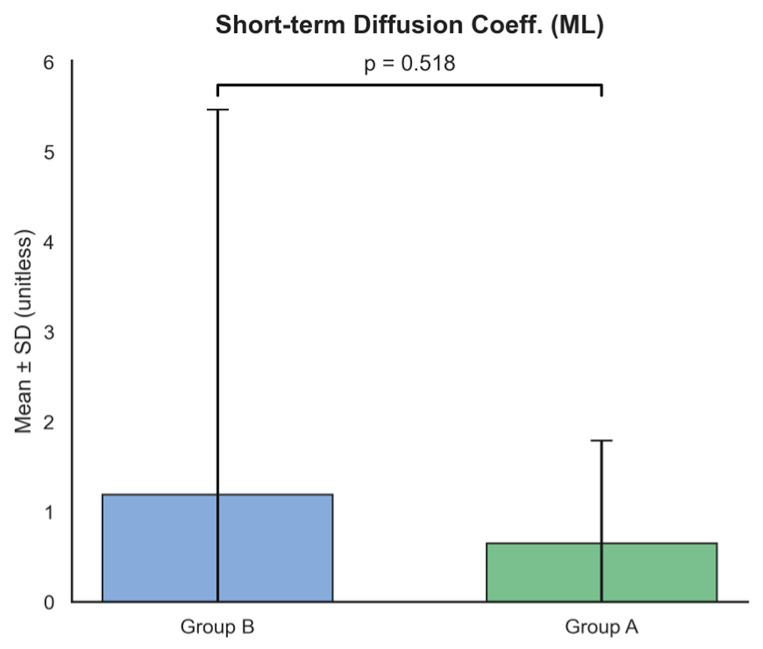
Comparison of stochastic features between Group A and Group B. The Short-term Diffusion Coefficient (ML) (unitless) shows no significant difference between groups (*p* = 0.518), suggesting that the underlying stochastic processes of postural sway remain consistent across cohorts. Data are expressed as Mean ± SD.

In the stochastic domain (Figure 4), the short-term diffusion coefficient in the mediolateral direction (short_time_diffusion_Diffusion_ML) did not differ significantly between groups (Group A: 0.65 ± 1.14 vs. Group B: 1.19 ± 4.28, *p* = 0.518). Neither short-term nor long-term diffusion coefficients showed significant group differences, suggesting no evident effect of mutation type on the stochastic characteristics of postural sway [28].

Descriptive and inferential statistics confirmed that CoP analysis identified one statistically significant difference in the frequency domain: the energy content above 2 Hz in the anteroposterior direction was significantly higher in Group A. Several other parameters exhibited trends but did not reach statistical significance. Further statistical details are presented in Supplementary Table S3.

## 3. Discussion

This study investigated the influence of *F8* genotype (INV vs. NonINV) on postural control strategies in patients with hemophilia A. The pivotal finding is that while the two groups were statistically comparable in terms of structural joint damage (Pettersson score) and macroscopic time-domain CoP parameters (positional and dynamic domains), a unique spectral signature emerged in the frequency domain. Specifically, the energy content above 2 Hz in the anteroposterior direction was the sole variable demonstrating statistical significance. The INV group (Group A) exhibited significantly higher energy in this frequency band compared to the NonINV group (Group B). This reveals that despite maintaining comparable gross postural performance, INV carriers utilize a distinct distal neuromuscular control strategy to maintain upright stability.

Unlike mediolateral (ML) stability, which is primarily governed by a hip strategy, anteroposterior (AP) postural stability relies predominantly on an ankle strategy [26,28]. Our observation of significantly elevated high-frequency energy (>2 Hz) in the AP direction among INV carriers strongly implicates a pathological mechanism concentrated within distal joint control loops. According to the inverted pendulum model, high-frequency, low-amplitude CoP oscillations are characteristic of increased joint stiffness utilized to maintain stability [29,30]. The elevated > 2 Hz energy likely reflects a state of muscular co-contraction or high-frequency micro-adjustments involving the tibialis anterior and gastrocnemius muscles [30]. This represents a quintessential “stiffening strategy,” aimed at freezing articular degrees of freedom to compensate for potential proprioceptive deficits or subclinical instability within the ankle joint [25].

In frequency domain analysis, low-frequency oscillations (<1 Hz) are typically associated with visual and vestibular integration, whereas frequencies above 2 Hz reflect proprioceptive reflex loops and the rapid modulation of muscle tension [31]. The anomalous elevation of energy in this frequency band among inversion participants suggests an aberration in sensory re-weighting. We hypothesize that chronic, microscopic damage to ankle mechanoreceptors—driven by the higher bleeding propensity associated with the inversion genotype—compels the central nervous system to increase the gain of the feedback loop [12,32]. This results in hyperactive reflexes and subsequent high-frequency oscillations [33]. Anatomically, the ankle joint is richly innervated by mechanoreceptors that are highly susceptible to recurrent hemarthrosis [1,32]. In patients with the INV genotype, who historically present with a more severe bleeding phenotype, subtle structural changes in the talocrural joint—potentially invisible on plain radiographs—may impair proprioceptive acuity [12]. Consequently, the central nervous system compensates by increasing lower-limb muscle stiffness through co-contraction of the tibialis anterior and triceps surae, manifesting as the observed high-frequency (>2 Hz) oscillations. While this mechanism successfully preserves global stability (explaining the lack of significant differences in positional parameters), it likely comes at the cost of increased metabolic expenditure and a heightened risk of neuromuscular fatigue.

The dissociation between the comparable Pettersson scores (Group A: 9.44 vs. Group B: 8.11, *p* > 0.05) and the distinct spectral CoP alterations underscores a critical insight: the functional impact of the Inversion genotype may precede radiographically visible osteochondral destruction [12,34]. Consequently, AP high frequency energy serves as a potential early warning biomarker, identifying inversion patients whose ankles appear structurally intact but have functionally entered a stage of “compensated stiffness” [18]. Furthermore, while ML deviations typically imply proximal (hip/trunk) deficits, our findings localize the instability specifically to the AP direction. This aligns precisely with clinical observations identifying the ankle as the primary target joint in hemophilia [1].

Several limitations of this study necessitate cautious interpretation. First, the cross-sectional design precludes the establishment of a direct causal pathway between *F8* mutation type and postural control strategies. The absence of longitudinal bleeding data and advanced imaging analysis makes it difficult to definitively distinguish whether the genotype influences postural control directly or operates indirectly through cumulative joint damage. Second, although strict inclusion criteria were applied to ensure genetic homogeneity, the relatively small sample size (*n* = 35) may limit the broader generalizability of our findings. Third, our assessment was confined to static bipedal stance, potentially overlooking balance deficits that manifest during dynamic activities; thus, future investigations should incorporate dynamic posturography or functional scales to better assess fall risk. Finally, potential confounding variables, including long-term prophylactic regimens, habitual physical activity, and proximal kinetics (hip, pelvis, and spine), were not controlled for. Given that recent studies have highlighted the critical impact of hip pathology [35,36] and avascular necrosis [37] on the kinetic chain and postural alignment, unmeasured proximal compensations could potentially confound the CoP results, even though our spectral findings primarily point towards a distal (ankle-steered) mechanism.

In conclusion, clinical assessment of inversion carriers should not be limited to radiographic imaging or static balance duration. The presence of high-frequency AP oscillations dictates a specific rehabilitation paradigm: priority must be given to proprioceptive training and fine motor control enhancement of the ankle joint, rather than generalized strengthening [32,38]. Mitigating this compensatory high-frequency stiffness may be crucial for preventing long-term articular degeneration and fatigue-induced injuries [39].

## 4. Materials and Methods

### 4.1. Study Design

A cross-sectional study was conducted to investigate balance control in individuals with variant gene mutations. The study protocol was approved by the institutional review board, and written informed consent was obtained from all participants.

### 4.2. Study Period

Data collection was performed over a nine-year period from March 2015 to 25 December 2024.

### 4.3. Participant Recruitment

Participants with hemophilia were recruited from Hemophilia Treatment and Thrombosis Center of Changhua Christian Hospital.

### 4.4. Inclusion and Exclusion Criteria

Participants were eligible for inclusion if they had a confirmed diagnosis of hemophilia, received routine follow-up and treatment at our institution, and had undergone genetic testing to identify the specific gene mutation type. Additional inclusion criteria required participants to have the ability to ambulate independently without assistive devices, possess intact cognitive function, and, in the case of pediatric participants, have informed consent provided by a legally authorized representative.

Exclusion criteria encompassed the presence of lower limb fractures, a history of total knee or hip arthroplasty, prior radiotherapy for lower limb joint synovitis, a documented history of brain injury, or the presence of active joint bleeding in the lower limbs as confirmed by ultrasound on the day of assessment. Participants were also excluded if they were unable to ambulate or maintain balance without assistive devices.

### 4.5. Participants Grouping by Genetic Type

All participants were categorized into two groups based on genetic analysis results: Group A (individuals with *INV22* or *INV1* mutations) and Group B (individuals with gene mutations other than inversion type).

### 4.6. Genetic Analysis Methods

Genetic screening for *F8* variants included inverse PCR (I-PCR) and multiplex PCR to detect common intron 22 and intron 1 inversions, as well as Sanger sequencing for exon and exon–intron junction variants. Variant nomenclature adhered to Human Genome Variation Society (HGVS) guidelines (https://www.hgvs.org/; accessed on 10 August 2022). Multiplex ligation-dependent probe amplification (MLPA) using SALSA MLPA Probemix P178 *F8* was conducted to identify deletions undetectable by PCR due to wild-type allele masking. MLPA-based exon dosage analysis followed previously described methods [40].

### 4.7. Data Collection

#### 4.7.1. Pettersson Score

Joint health was assessed using the Pettersson radiological score, which evaluates joint damage in hemophilia patients. Radiographic assessment was performed annually with standardized full-joint X-rays. Two experienced physicians independently reviewed all radiographs and assigned Pettersson scores according to established criteria. In cases of disagreement, the two readers held a consensus meeting to discuss and determine a final score. Inter-rater reliability was maintained through regular calibration sessions, ensuring consistent scoring across assessments.

#### 4.7.2. Center of Pressure (CoP)

**Instruments.** CoP measurements were obtained using a stabilographic platform (Mullitrix; Diasu, Rome, Italy). CoP data were recorded at a sampling frequency of 20 Hz over a 50 s period. The raw signals were then pre-processed using a fourth-order low-pass Butterworth filter with a 10 Hz cutoff frequency to reduce high-frequency noise. Processed CoP data were subsequently analyzed using open-source software tools as previously described preprocessed CoP signals were analyzed using open-access code.

**Procedures.** Participants stood barefoot without braces on the platform in a relaxed, natural position with arms hanging loosely at their sides and eyes open. Each participant performed three trials with 2 min rest intervals between measurements to minimize fatigue effects. Room temperature and lighting conditions were standardized across all testing sessions.

**Data Preprocessing.** The collected CoP data were initially preprocessed using a fourth-order, low-pass Butterworth filter with a 10 Hz cutoff frequency to remove high-frequency noise and artifacts. The preprocessed CoP signals were subsequently analyzed using validated open-access algorithms across multiple analytical domains:

**Positional Variables.** These variables focus on the spatial distribution of the CoP trajectory, capturing characteristics such as dispersion, average position, or the geometric shape of the stabilogram. They are used to assess static postural stability and are independent of time dynamics.

**Dynamic Variables.** These variables analyze the time-varying aspects of the CoP trajectory, including displacement velocity, acceleration, or dynamic behavior. They reflect the kinematic adjustments of the postural control system during balance maintenance.

**Frequency Variables.** These variables examine the spectral characteristics of the CoP trajectory through frequency domain analysis, such as Fourier transforms. The CoP signal was decomposed into three functional frequency bands representing distinct postural strategies. The low-frequency band (<0.5 Hz) captures slow, drift-like movements associated with visuo-vestibular integration, where elevated power indicates a heightened reliance on visual or vestibular inputs for sensory processing. In contrast, the medium-frequency band (0.5–2.0 Hz) corresponds to the ankle strategy, reflecting the inverted pendulum mechanism and the effective regulation of lower limb stiffness via proprioceptive feedback from the gastrocnemius and soleus muscles. Finally, the high-frequency band (>2.0 Hz) encompasses rapid corrective oscillations associated with the hip strategy or compensatory stiffening. An increase in energy content within this high-frequency domain signals a departure from the efficient ankle strategy, suggesting that the postural control system is responding to instability or proprioceptive deficits through rapid torque adjustments at proximal joints or increased muscle co-contraction.

**Stochastic Variables.** These variables are based on random process theory, assessing the stochastic properties of the COP trajectory, such as diffusion or random walk behavior. They evaluate long-term stability and predictability in postural control.

**Figure 5:**
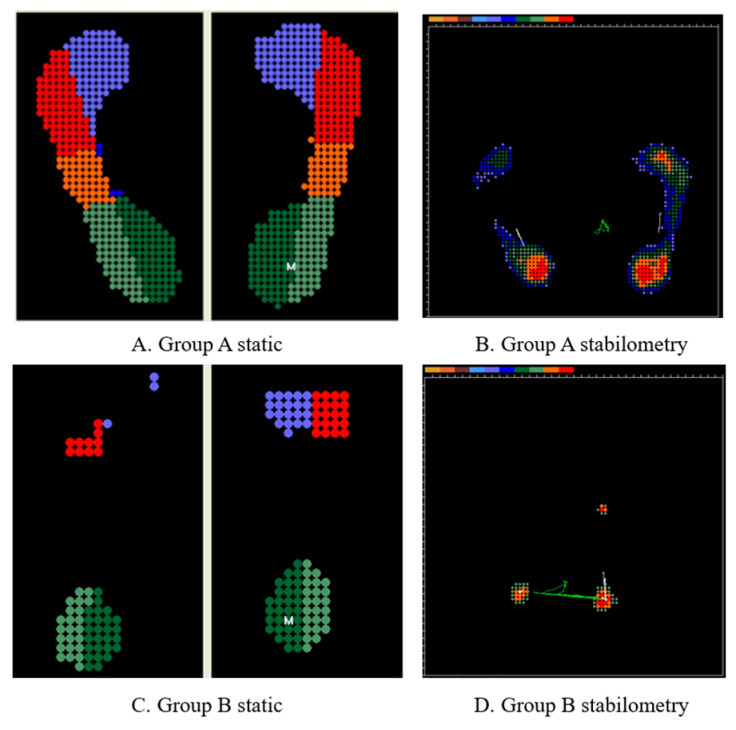
Conceptual illustration of postural stability patterns in hemophilia patients with distinct mutation types. (**A**,**B**) Representative center of pressure (CoP) distribution in a Group A participant, demonstrating a more dispersed and asymmetrical pressure pattern, suggestive of impaired postural control. (**C**,**D**) Representative CoP distribution in a Group B participant, characterized by a more centralized and stable footprint and sway trajectory. In the static footprints (**A**,**C**), the color gradient represents relative pressure distribution, where warmer colors (red/orange) indicate areas of peak pressure and cooler colors (blue/green) indicate lower pressure zones. In the stabilometry plots (**B**,**D**), green lines illustrate the CoP sway trajectory. The letter “M” denotes the calculated midpoint of the pressure distribution for each foot.4.8. Statistical Analysis.

Based on previous studies, we hypothesized that individuals with Group A are more prone to joint bleeding, which may adversely affect postural balance. Consequently, greater variability in CoP measures during quiet standing is expected in this group. The findings illustrated in Figure 5 align with this hypothesis, demonstrating increased CoP variability in a participant in Group A.

Statistical analyses were conducted using Python (version 3.12.7; Anaconda, Inc., Austin, TX, USA) within the Jupyter Notebook environment (Version 7.2.2). Differences between groups categorized by genetic variant type were evaluated for each parameter using Mann–Whitney *U* tests, with statistical significance set at *p* < 0.05. Group means ± standard deviations (SD) were also calculated and reported to convey the direction and magnitude of differences. Each parameter was compared between groups adjusted for age. Given the limited sample size and the novel nature of this investigation, the statistical approach was considered exploratory. Therefore, correction for multiple comparisons was not strictly applied to avoid Type II errors in this preliminary identification of genotype-specific biomarkers. All analyses proceeded through automated, loop-based code to ensure consistency and reproducibility.

## 5. Conclusions

This study found an association between *F8* mutation type (INV vs. NonINV) and postural control strategies in Taiwanese hemophilia A patients. While the cross-sectional design limits causal inference, CoP analysis revealed genotype-specific postural differences. Larger, multi-center longitudinal studies are needed to validate and generalize these findings.

## Figures and Tables

**Table 1:** Demographic Characteristics.

Basic Characteristics	All (*n* = 35)			Group A (*n* = 16)			Group B (*n* = 19)			*p*-Value
Age (year)	23.61	±	15.55	21.32	±	14.35	25.54	±	16.63	0.48
Height (cm)	159.23	±	23.95	153.33	±	25.74	164.20	±	21.79	0.20
Weight (kg)	60.52	±	25.15	53.87	±	23.09	66.12	±	26.04	0.18
BMI	22.44	±	5.10	21.33	±	4.00	23.38	±	5.82	0.52
Pettersson score of lower limbs	8.71	±	10.40	9.44	±	12.34	8.11	±	8.74	0.96
Inhibitor (Positive)	3 (8.6%)			2 (12.5%)			1 (5.3%)			0.58

**Table 2:** Distribution of Variant Types in Participants with Hemophilia A (*n* = 35).

Variant Type	Number (%)
*INV22/INV1* (Group A)	15/1 (45.7)
Mutations other than INV (Group B)	19 (54.3)
Missense	11 (31.4)
Small Deletion	4 (11.4)
Splice site mutation	1 (2.9)
Nonsense	1 (2.9)
Large Deletion	1 (2.9)
Duplication	1 (2.9)

Note: INV, inversion.

## Data Availability

The data generated and analyzed in this study are not publicly available due to patient confidentiality but may be available from the corresponding author upon reasonable request and with appropriate ethical approvals.

## Supplementary Materials

The following supporting information can be downloaded at: https://www.mdpi.com/article/10.3390/ijms27052323/s1.

## Abbreviations

CoP	Center of pressure
INV	Inversion
*F8*	Factor VIII
SD	Standard deviation
IRB	Institutional review board
