# Supplementary material for: Genotype-Specific Postural Control Deficits in Hemophilia A: Insights from Center of Pressure Analysis Beyond Radiographic Arthropathy
Source: Int J Mol Sci. 2026 Mar 1;27(5):2323. doi: 10.3390/ijms27052323 (PMC12986132; doi:10.3390/ijms27052323)
Supplement: Supplementary file 1 [file ijms-27-02323-s001.zip › ijms-4133626-supplementary.pdf]

Supplementary

Table S1. Patient Demographics and Clinical Characteristics

No	Age	Variant type	Height (cm)	Weight (kg)	BMI (kg/m²)	Inhibitor
01	29Y10M	INV22	178.4	70.2	22.1	Negative
02	42Y2M	INV22	178.0	77.6	24.5	Negative
03	11Y0M	INV22	140.0	40.8	20.8	Negative
04	22Y4M	INV22	181.3	84.6	25.7	Negative
05	10Y3M	INV1	133.4	31.7	17.8	Negative
06	11Y3M	INV22	143.0	49.8	24.4	Negative
07	17Y8M	INV22	176.0	80.0	25.8	Negative
08	20Y4M	INV22	168.4	60.7	21.4	Negative
09	50Y11M	INV22	176.4	71.6	23.0	Negative
10	34Y5M	INV22	164.0	67.0	24.9	Positive
11	5Y4M	INV22	110.0	17.0	14.0	Negative
12	24Y1M	INV22	168.0	72.0	25.5	Negative
13	5Y9M	INV22	109.6	19.2	16.0	Positive
14	5Y1M	INV22	115.7	18.8	14.0	Negative
15	11Y6M	INV22	141.1	40.9	20.5	Negative
16	39Y3M	INV22	170.0	60.0	20.8	Negative
17	7Y11M	Duplication	116.8	24.0	17.6	Positive
18	16Y5M	Missense	165.6	54.0	19.7	Negative

19	27Y7M	Nonsense	173.0	90.0	30.1	Negative
20	23Y9M	Splice site mutation	176.9	111.0	35.5	Negative
21	28Y1M	Small Deletion	177.6	113.0	35.8	Negative
22	50Y6M	Missense	168.8	73.0	25.6	Negative
23	60Y1M	Missense	178.0	69.0	21.8	Negative
24	13Y3M	Large Deletion	159.7	45.6	17.9	Negative
25	5Y8M	Missense	120.0	30.0	20.8	Negative
26	14Y6M	Missense	161.0	47.0	18.1	Negative
27	16Y0M	Missense	170.0	65.0	22.5	Negative
28	5Y0M	Small Deletion	116.1	18.7	13.9	Negative
29	48Y9M	Missense	180.0	81.0	25.0	Negative
30	15Y3M	Missense	173.3	75.6	25.2	Negative
31	36Y0M	Missense	179.5	82.1	25.5	Negative
32	18Y7M	Small Deletion	177.6	63.8	20.2	Negative
33	13Y0M	Missense	165.2	54.4	19.9	Negative
34	38Y0M	Missense	186.0	74.9	21.6	Negative
35	47Y0M	Small Deletion	174.7	84.1	27.6	Negative

Note: INV22, inversion 22; Age is presented as Years (Y) and Months (M).

Table S2. The Pettersson score of lower limbs among all participants

	Ankle Rt	Ankle Lt	Knee Rt	Knee Lt	Hip Rt	Hip Lt	Totals
Group A							
01	0	6	1	1	0	2	10
02	13	13	0	0	0	0	26
03	0	0	0	0	0	0	0
04	0	0	9	9	0	0	18
05	0	0	0	0	0	0	0
06	0	0	0	0	0	0	0
07	0	0	3	0	2	2	7
08	0	0	0	0	0	0	0
09	10	12	10	0	0	4	36
10	4	13	10	0	0	0	27
11	0	0	0	0	0	0	0
12	0	0	0	0	1	1	2
13	0	2	0	0	0	0	2
14	0	0	0	0	0	0	0
15	0	0	0	0	0	0	0
16	11	10	0	0	1	1	23
Group B							
17	3	3	9	0	0	0	15
18	0	0	1	1	2	2	6
19	9	2	0	0	0	0	11
20	8	0	0	5	0	0	13
21	10	0	2	6	2	2	22
22	0	0	10	0	0	0	10
23	10	0	0	0	1	1	12
24	0	0	0	0	0	0	0
25	0	0	0	0	0	0	0
26	0	7	0	0	0	0	7
27	7	0	0	0	0	0	7
28	0	0	0	0	0	0	0
29	9	9	0	0	1	1	20
30	0	0	0	0	0	0	0
31	0	0	0	0	0	0	0
32	0	0	0	0	0	0	0
33	0	0	0	0	2	0	2
34	0	0	0	0	0	0	0
35	12	9	2	2	2	2	29

Table S3. Statistical details of CoP variables.

Variable	Group A	Group B	Beta	SE	P-value
Mean Position (ML) (cm)	0.01 ± 0.00	0.01 ± 0.00	0.00	0.00	0.69
Mean Position (AP) (cm)	0.00 ± 0.00	0.00 ± 0.00	0.00	0.00	0.42
Mean Distance (ML) (cm)	0.46 ± 0.47	0.34 ± 0.34	0.11	0.14	0.42
Mean Distance (AP) (cm)	0.52 ± 0.42	0.43 ± 0.25	0.09	0.12	0.48
Mean Distance (Radius) (cm)	0.76 ± 0.61	0.63 ± 0.38	0.12	0.17	0.48
Maximal Distance (ML) (cm)	1.67 ± 1.23	1.13 ± 1.14	0.53	0.41	0.20
Maximal Distance (AP) (cm)	1.77 ± 1.22	1.36 ± 0.83	0.43	0.36	0.23
Maximal Distance (Radius) (cm)	2.33 ± 1.52	1.83 ± 1.18	0.49	0.47	0.30
Rms (ML)	0.59 ± 0.57	0.43 ± 0.45	0.15	0.18	0.39
Rms (AP)	0.66 ± 0.50	0.54 ± 0.32	0.12	0.14	0.42
Rms Radius	0.92 ± 0.71	0.75 ± 0.46	0.16	0.20	0.44
Range (ML)	2.78 ± 2.28	1.98 ± 2.13	0.76	0.76	0.33
Range (AP)	2.86 ± 1.85	2.38 ± 1.63	0.52	0.60	0.40
Range (Total)	3.77 ± 2.63	3.18 ± 2.23	0.55	0.84	0.52
Range Ratio (Total)	1.58 ± 1.92	1.15 ± 1.60	0.28	0.58	0.63
Planar Deviation (Total)	0.92 ± 0.71	0.75 ± 0.46	0.16	0.20	0.44
Coefficient Sway Direction (Total)	-0.13 ± 0.54	0.04 ± 0.63	-0.17	0.21	0.41
Confidence Ellipse Area (cm²)	6.24 ± 8.26	3.96 ± 6.42	2.24	2.54	0.39
Principal Sway Direction (Total)	42.20 ± 25.90	27.83 ± 25.84	13.74	8.96	0.14
Mean Velocity (ML) (cm/s)	1.16 ± 0.53	0.99 ± 1.09	0.12	0.30	0.68
Mean Velocity (AP) (cm/s)	0.89 ± 0.43	0.83 ± 0.34	0.07	0.13	0.60
Mean Velocity (Total) (cm/s)	1.62 ± 0.67	1.48 ± 1.06	0.10	0.31	0.74
Sway Area Per Second (Total)	0.44 ± 0.51	0.25 ± 0.28	0.19	0.14	0.18
Phase Plane Parameter (ML)	1.91 ± 1.00	1.56 ± 2.01	0.28	0.56	0.62
Phase Plane Parameter (AP)	1.63 ± 0.93	1.31 ± 0.54	0.33	0.26	0.21
Lfs (Total)	21.76 ± 51.59	13.40 ± 8.63	6.92	12.16	0.57
Fractal Dimension (Total)	1.75 ± 0.24	1.77 ± 0.19	-0.04	0.07	0.59
Zero Crossing Spd (ML)	166.94 ± 107.30	142.84 ± 61.40	25.31	29.66	0.40
Peak Velocity Pos Spd (ML)	1.33 ± 0.54	1.14 ± 1.15	0.15	0.32	0.64
Peak Velocity Neg Spd (ML)	1.47 ± 0.68	1.31 ± 1.69	0.09	0.45	0.85
Peak Velocity All Spd (ML)	1.40 ± 0.60	1.23 ± 1.42	0.12	0.39	0.76
Zero Crossing Spd (AP)	163.62 ± 138.77	120.84 ± 46.39	40.14	34.49	0.25
Peak Velocity Pos Spd (AP)	1.14 ± 0.74	0.95 ± 0.37	0.20	0.20	0.33
Peak Velocity Neg Spd (AP)	1.00 ± 0.47	0.93 ± 0.38	0.07	0.15	0.65
Peak Velocity All Spd (AP)	1.07 ± 0.59	0.94 ± 0.38	0.13	0.17	0.44
Mean Peak Sway Density	3.33 ± 7.27	1.84 ± 0.80	1.36	1.71	0.43

Mean Distance Peak Sway Density	0.44 ± 0.28	0.42 ± 0.40	0.00	0.12	0.99
Mean Frequency (ML)	0.56 ± 0.22	0.63 ± 0.25	-0.07	0.08	0.41
Mean Frequency (AP)	0.42 ± 0.24	0.38 ± 0.12	0.03	0.06	0.61
Mean Frequency (Total)	0.41 ± 0.15	0.40 ± 0.12	0.01	0.05	0.82
Total Power (ML)	3.16 ± 5.65	1.77 ± 3.22	1.50	1.56	0.34
Total Power (AP)	3.30 ± 4.59	2.60 ± 5.80	0.77	1.84	0.68
Power Frequency 50 (ML)	0.37 ± 0.11	0.33 ± 0.08	0.05	0.03	0.13
Power Frequency 50 (AP)	0.31 ± 0.09	0.29 ± 0.07	0.01	0.03	0.63
Power Frequency 95 (ML)	1.52 ± 0.58	1.41 ± 0.60	0.14	0.20	0.51
Power Frequency 95 (AP)	1.28 ± 0.73	0.96 ± 0.40	0.31	0.20	0.13
Frequency Mode (ML) (Hz)	0.22 ± 0.10	0.25 ± 0.09	-0.03	0.03	0.33
Frequency Mode (AP) (Hz)	0.20 ± 0.04	0.21 ± 0.06	-0.01	0.02	0.55
Centroid Frequency (ML)	0.77 ± 0.21	0.74 ± 0.19	0.04	0.07	0.57
Centroid Frequency (AP)	0.67 ± 0.26	0.54 ± 0.14	0.12	0.07	0.11
Frequency Dispersion (ML)	0.71 ± 0.05	0.73 ± 0.06	-0.02	0.02	0.31
Frequency Dispersion (AP)	0.70 ± 0.06	0.68 ± 0.06	0.02	0.02	0.30
Energy Content < 0.5 Hz (ML) (cm^2)	2.48 ± 4.96	1.49 ± 2.85	1.08	1.37	0.44
Energy Content < 0.5 Hz (AP) (cm^2)	2.82 ± 4.22	2.34 ± 5.46	0.52	1.72	0.76
Energy Content 0.5–2 Hz (ML) (cm^2)	0.62 ± 0.88	0.26 ± 0.37	0.39	0.23	0.10
Energy Content 0.5–2 Hz (AP) (cm^2)	0.44 ± 0.48	0.25 ± 0.34	0.22	0.14	0.13
Energy Content > 2 Hz (ML) (cm^2)	0.06 ± 0.06	0.03 ± 0.05	0.03	0.02	0.16
**Energy Content > 2 Hz (AP) (cm^2)**	0.04 ± 0.05	0.01 ± 0.01	0.03	0.01	0.03
Frequency Quotient (ML)	0.04 ± 0.03	0.03 ± 0.02	0.00	0.01	0.75
Frequency Quotient (AP)	0.03 ± 0.05	0.01 ± 0.01	0.02	0.01	0.17
Short-term Diffusion Coeff. (ML)	0.65 ± 1.14	1.19 ± 4.28	-0.72	1.11	0.52
Long-term Diffusion Coeff. (ML)	0.91 ± 1.63	0.76 ± 2.34	0.10	0.71	0.89
Critical Time (ML) (s)	1.83 ± 2.86	1.31 ± 0.99	0.56	0.72	0.44
Critical Displacement (ML)	0.88 ± 1.38	0.38 ± 1.09	0.53	0.43	0.22
Short Time Scaling (ML)	0.64 ± 0.11	0.63 ± 0.13	0.01	0.04	0.80
Long Time Scaling (ML)	0.11 ± 0.29	0.15 ± 0.40	-0.03	0.12	0.78
Short-term Diffusion Coeff. (AP)	0.58 ± 0.66	0.41 ± 0.41	0.18	0.19	0.34
Long-term Diffusion Coeff. (AP)	0.62 ± 0.90	0.81 ± 1.46	-0.23	0.43	0.59
Critical Time (AP) (s)	1.60 ± 1.53	1.05 ± 0.85	0.52	0.42	0.23
Critical Displacement (AP)	2.94 ± 8.10	0.59 ± 1.03	2.38	1.92	0.22
Short Time Scaling (AP)	0.68 ± 0.16	0.75 ± 0.09	-0.06	0.04	0.15
Long Time Scaling (AP)	0.26 ± 0.35	0.09 ± 0.47	0.16	0.15	0.27
